# Micron-Sized SiO_x_-Graphite Compound as Anode Materials for Commercializable Lithium-Ion Batteries

**DOI:** 10.3390/nano12121956

**Published:** 2022-06-07

**Authors:** Minki Jo, Soojin Sim, Juhyeong Kim, Pilgun Oh, Yoonkook Son

**Affiliations:** 1 Department of Electric Engineering, Chosun University, 309, Pilmun-daero, Dong-gu, Gwangju 61452, Korea; mkjo@chosun.ac.kr (M.J.); juhyeong@chosun.kr (J.K.); 2 Department of Energy Engineering, School of Energy and Chemical Engineering, Ulsan National Institute of Science and Technology (UNIST), Ulsan 44919, Korea; thegreatest25@gmail.com; 3 Department of Smart Green Technology Engineering, Pukyong National University, Busan 485471, Korea; poh@pknu.ac.kr; 4 Department of Nanotechnology Engineering, Pukyong National University, Busan 485471, Korea

**Keywords:** SiO_x_, SiO_x_-graphite compound, anode materials, lithium-ion batteries

## Abstract

The electrode concept of graphite and silicon blending has recently been utilized as the anode in the current lithium-ion batteries (LIBs) industry, accompanying trials of improvement of cycling life in the commercial levels of electrode conditions, such as the areal capacity of approximately 3.3 mAh/cm^2^ and volumetric capacity of approximately 570 mAh/cm^3^. However, the blending concept has not been widely explored in the academic reports, which focused mainly on how much volume expansion of electrodes could be mitigated. Moreover, the limitations of the blending electrodes have not been studied in detail. Therefore, herein we investigate the graphite blending electrode with micron-sized SiO_x_ anode material which is one of the most broadly used Si anode materials in the industry, to approach the commercial and practical view. Compared to the silicon micron particle blending electrode, the SiO_x_ blending electrode showed superior cycling performance in the full cell test. To elucidate the cause of the relatively less degradation of the SiO_x_ blending electrode as the cycling progressed in full-cell, the electrode level expansion and the solid electrolyte interphase (SEI) thickening were analyzed with various techniques, such as SEM, TEM, XPS, and STEM-EDS. We believe that this work will reveal the electrochemical insight of practical SiO_x_-graphite electrodes and offer the key factors to reducing the gap between industry and academic demands for the next anode materials.

## 1. Introduction

The growing demand for electric vehicles has sparked great interest in lithium-ion batteries (LIBs) with high energy density for a longer driving range. To meet the higher energy density of LIBs, various electrode materials and formats have been introduced. Especially for the anode materials, Si has many attractive advantages such as its approximately 10 times larger theoretical capacity compared to graphite (372 mAh/g), comparatively low reduction potential, and abundance on earth [1,2,3,4,5]. However, unlike graphite, which exhibits only a capacity decrease of less than 30% during 2000 cycles despite full cell evaluation under typical electrolyte conditions [6], the volume expansion of Si and the solid electrolyte interphase (SEI) formation derived by decomposition of non-aqueous liquid electrolyte during lithiation lead to rapid and gradual capacity degradation of Si anode in cycling life [7,8]. Moreover, under commercial electrode conditions based on the graphite anode, such as the areal capacity of 3.3 mAh/cm^2^ (loading level: 10 mg/cm^2^) and volumetric capacity of 570 mAh/cm^3^ (electrode density: 1.6 g/cc), the cell degradation of the Si anode induced by volume expansion in the electrode arises more severely [9,10]. To avoid the fading mechanisms in Si anode, diverse strategies have been innumerably reported [11]. For example, morphological design providing void space is one of the most well-known strategies, and representative designs include hollow nanoparticles (2725 mAh/g initial capacity and 52% capacity retention after 700 cycles), nanotubes (1780 mAh/g initial capacity and 88% capacity retention after 6000 cycles) and yolk-shells (2833 mAh/g initial capacity and 74% capacity retention after 1000 cycles) [7,12,13,14,15]. Another familiar strategy is forming a robust buffer matrix with various materials, such as carbon (1950 mAh/g initial capacity and ~100% capacity retention after 100 cycles) and metal oxide materials (~1000 mAh/g initial capacity and ~65% capacity retention after 1000 cycles) [16,17,18,19]. Beyond the physical/chemical material design of Si, Si-graphite-blending-based materials were recently introduced to compensate for the limitation of Si via diluting its content in the anode. This strategy has the advantage to overcome the difficulties of Si anode via an easy and simple blending approach. Therefore, currently, up to 5 wt% Si mixed with graphite anodes is utilized for commercial anodes, satisfying the harsh commercial electrode conditions [9]. The roles of graphite in Si-graphite anode are alleviating the volume expansion, enhancing electrical conductivity, and increasing the packing density of the electrode [10]. In this regard, practical investigation of Si-graphite anode with commercial loading level and electrode density is a significant field of academic and industrial battery society to increase the content of Si in anode for higher energy density.

Among the diverse approaches for Si materials design, SiO_x_ is one of the most widely used designs because of its great cycle stability and scalable synthetic routes [20,21,22]. The SiO_x_ anode shows stable cycling life, although it has low initial coulombic efficiency (ICE) with the intrinsic problem. The low ICE of the SiO_x_ anode is caused via the formation of lithium dioxide (Li_2_O) or lithium silicate (usually, Li_4_SiO_4_ or Li_2_Si_2_O_5_) [23]. Lowering the oxygen content in SiO_x_ reduces the formation of Li_2_O, Li_4_SiO_4_, and Li_2_Si_2_O_5_. However, Li_2_O and lithium silicate have also been supposed as robust buffer matrices to improve the cycling performance of SiO_x_ anode [24,25]. Hereafter, oxygen content in SiO_x_ anode would be a trade-off relationship between ICE and cycle performance. To study the potential beyond this limitation of SiO_x_ anode as a practical approach for the LIBs industry, the graphite blending concept is proposed in this study. In the graphite blending concept, graphite not only reduces the expansion of the electrode but also improves the electronic conductivity of the electrode. Here, we prepared the blending electrode of SiO_x_ and graphite to gravimetric and areal capacities of 440 mAh/g and 3.5 mAh/cm^2^, respectively. The electrode density of the blending electrode was approx.1.6 g/cc, and a minimum amount of 3 wt% binders was utilized. We investigated the detailed degradation mechanisms of SiO_x_-graphite blending anode in full cells with reliable ex-situ analyses, such as SEM, TEM, and XPS. We believe that our findings will be helpful to analyze the degradation mechanism of Si-graphite anode for LIBs and enlighten the considerations of future practical use of Si-graphite anode for containing more higher ratio of Si.

## 2. Materials and Methods

All electrode materials were purchased from commercial battery materials incorporation. To achieve the gravimetric capacity of 440 mAh/g, the blending ratios of SiO_x_: natural graphite = 6.9:93.1 (*w*/*w*) for SiO_x_ blending and silicon micron particles (SiMPs): natural graphite = 4.0:96.0 (*w*/*w*) for SiMPs were utilized. The SiO_x_ and SiMPs blending electrodes consisted of active material: carboxymethyl cellulose (CMC): styrene-butadiene rubber (SBR) = 97:1.5:1.5 (*w*/*w*) were prepared. For the full cell evaluations, the cathode electrodes using NCM622 cathode material were prepared with electrode ratio of active material, super-P, and PVDF = 94:3:3. For the direct comparison of all electrodes, the loading levels of all anodes were adjusted to achieve approx. 3.5 mAh/cm^2^. For the used electrolyte, 1.3M LiPF_6_ was dissolved in ethylene carbonate (EC), ethyl-methyl carbonate (EMC), and dimethyl carbonate (DEC) which had a volume ratio of 3:5:2 with the addition of 0.2 wt% LiBF_4_, 10.0 wt% fluoroethylene carbonate (FEC), and 0.5 wt% vinylene carbonate (VC) (Panax Etec). A microporous polyethylene (PE) (Celgard) was used as a separator.

For the half-cell test, punched anodes of a diameter of 15 mm were used. All the cells were assembled with 2032 coin-type cells in an argon-filled glove box. For the formation of cells, 2 cycles of charging and discharging were performed at 0.2 C. Cycling life evaluation was performed with charging and discharging at 0.5 C. The cut-off voltage of 0.01–1.5 V for constant current mode (CC mode) and cut-off C-rate of 0.02 C for constant voltage (CV mode) were utilized. For the full-cell test, punched anodes of a diameter of 15 mm and punched cathodes of a diameter of 14 mm were used. The N/P ratio of full cells was 1.04 ± 0.1. As for the formation condition of full-cell, 3 cycles were performed with charging and discharging at 0.2 C. The cycle condition of the full-cell was 0.5 C charging and discharging for 500 cycles. The cut-off conditions of CC and CV modes were 4.4–2.75 V and 0.02 C-rate, respectively, in the full-cell test.

The morphology of the samples was observed using scanning electron microscopy (SEM) (Verios 460, FEI). The transmission electron microscopy (TEM) specimens were prepared using a focused ion beam (FIB, Quanta 3D FEG, FEI). The TEM images and high-resolution TEM images were captured using a JEOL JEM-2100 that was operated at 200 kV. Scanning transmission electron microscope (STEM) bright-field (BF) images and STEM- energy-dispersive X-ray spectroscopy (EDS) scan were operated with Oxford Aztec TEM. X-ray diffraction (XRD) patterns were obtained using a Rigaku D/Max-2200/PC and investigated with Cu-Kα radiation at 40 kV and 40 mA in a step of 0.02 degrees. The X-ray photoelectron spectroscopy (XPS) was operated using a Thermo Fisher K-alpha radiation of energy beam (1486.6 eV). Binding energies of all elements were calibrated concerning the C 1s peak at 284.4 eV. To analyze the core part of active materials, depth profiling was conducted under conditions of 1 keV Ar^+^ for 10 min in XPS.

## 3. Results and Discussion

**Figure 1:**
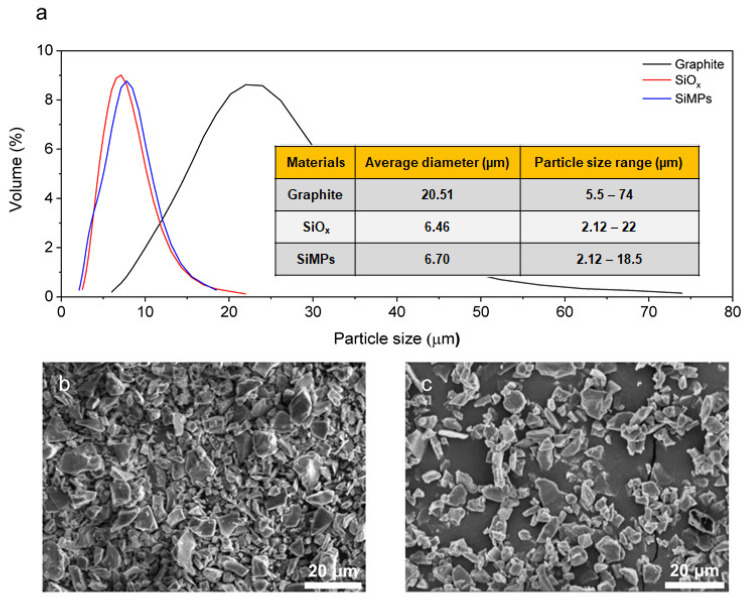
(**a**) Particle size distribution of graphite, SiO_x_, and SiMPs. SEM images of (**b**) SiMPs and (**c**) SiO_x_.

The micron-sized SiO_x_ particles have an average diameter of 6.46 μm shown in Figure 1. Note that the size and distribution of SiMPs are similar to that of SiO_x_ particles. The SiO_x_ was a totally amorphous phase in Figure S1, which is dissimilar to preceding analysis reports on SiO_x_ structure which has a separated phase of crystalline Si and SiO_2_ [26]. Amorphous Si structure could be more promising than crystalline Si structure since Si-Si bond breaking in amorphous Si structure shows more stable fracture rearrangement behavior during lithiation regarding the formation cycle [27]. The chemical composition of SiO_x_ was investigated with the X-ray photoelectron spectroscopy (XPS) spectrum to confirm the different oxidation states of Si atoms in SiO_x_ depending on the depth in Figure S2. The Si 2p peaks corresponded to Si^4+^ (103.6 eV), Si^3+^ (102.5 eV), Si^2+^ (101.4 eV), and Si^0^ (99.4 eV), respectively [28]. At both surface and core of SiO_x_, Si^4+^ represents the highest major peak intensity. The other major peaks were Si^0^ and Si^2+^. The O 1s peak (532.9 eV) corresponded to the oxidation state in SiO_2_, and the other peaks (531.15 and 531.84 eV) represent the SiO_x_ [29]. From the surface to the core, in the O 1s spectrum, it was confirmed that the peak shift to lower binding energy is due to the characteristics of SiO_x_ rather than that of SiO_2_. In the Si 2p spectrum, from the surface to the core, it was observed that the peak shift representing Si^4+^ toward low binding energy and the characteristics of low Si oxidation peaks were remarkable due to the decrease in the oxidation state of Si. In addition, Si and SiO_2_ were found to be well mixed in the SiO_x_ phase despite native SiO_2_ on the surface [30].

**Figure 2:**
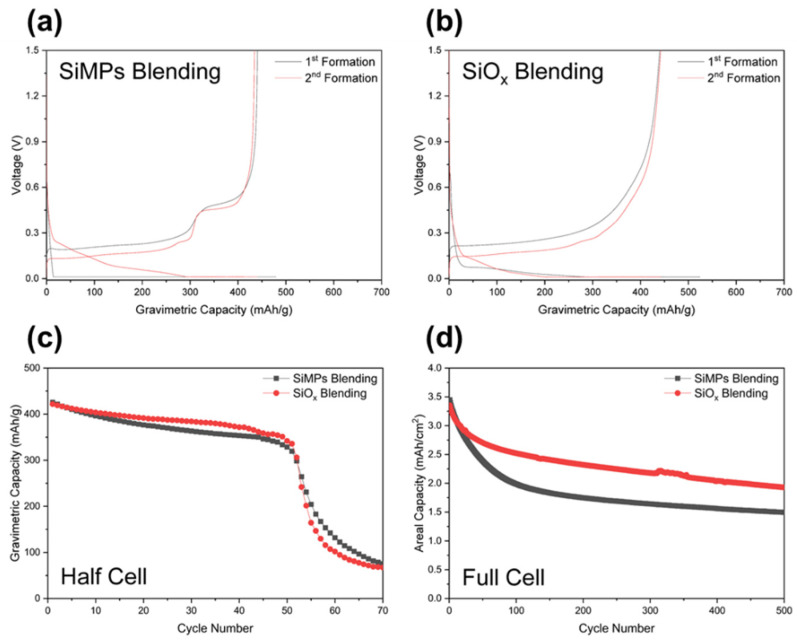
Half-cell formation voltage profiles of (**a**) SiMPs blending and (**b**) SiO_x_ blending. Comparison of cycling performance of SiMPs blending and SiO_x_ blending in (**c**) half-cell and (**d**) full-cell.

Figure 2a,b show half-cell formation voltage profiles of SiMPs and SiO_x_ blending electrodes, respectively. The first delithiation capacity showed approximately 440 mAh/g for both blending electrodes and the initial coulombic efficiency (ICE) of SiMPs and SiO_x_ blending electrodes were 92% and 84%, respectively. The voltage profile of SiMPs blending shows the voltage plateau of Li-Si redox reaction at 0.42 V and that of SiO_x_ blending shows no plateau which is typical in the previous SiO_x_ anode report [31,32]. Figure 2c,d show the cycling performance of SiMPs and SiO_x_ blending electrodes in half and full cells. In the half-cell test, both electrodes showed similar stable cycling stability up to the 50th cycle and the capacities rapidly dropped after the 50th cycle because of fading mechanisms on Li metal due to exhaustion of electrolyte and solid electrolyte interphase (SEI) thickening [33,34,35]. Since the half cells tests were limited to only 50 cycles, we conducted a full-cell test. As shown in Figure S3, the first discharge capacities and ICE of SiMPs and SiO_x_ blendings in full-cell were 3.54 mAh/cm^2^ and 89% for SiMPs blending and 3.45 mAh/cm^2^ and 80% for SiO_x_ blending. Note that areal loading and full cell design were adjusted to obtain a similar level of areal capacities. The cycling retentions of SiMPs and SiO_x_ blending electrodes at the 200th cycle in full-cell were 51% and 70%, respectively, and at the 500th cycle 29% and 56%, respectively. This indicates that SiO_x_ is more suitable than SiMPs in practical graphite blending electrodes.

**Figure 3:**
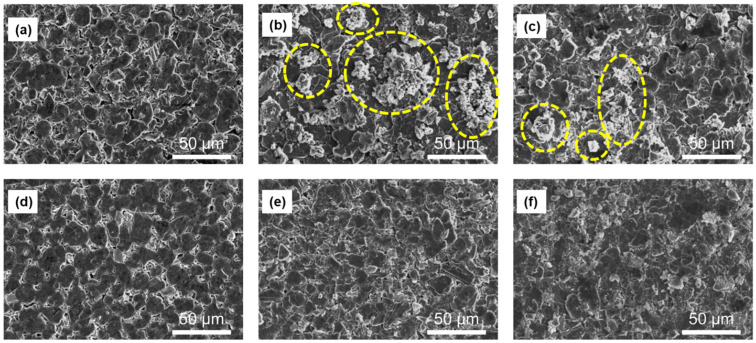
Top-view SEM images of (**a**–**c**) SiMP blending and (**d**–**f**) SiO_x_ blending electrodes in full-cell: (**a**) pristine, (**b**) after 250 cycles, (**c**) after 500 cycles, respectively.

We also measured the extent of swelling of the blending electrodes depending on the full cell cycling in Figure S4. All the samples were measured at a fully discharged state by a micrometer. The thickness of the SiO_x_ blending electrode expanded from 75 μm to 77 μm (after 250 cycles) and 90 μm (after 500 cycles), representing 103% and 120% expansion rates relative to the initial thickness, respectively. In contrast, the thickness of SiMP blending electrodes swelled from 63 μm to 122 μm (after 250 cycles) and 125 μm (after 500 cycles), representing 194% and 198% expansion rates relative to the initial thickness, respectively. As shown in Figure 3, there were no serious cracks in the electrodes due to volume expansion in both blending samples during the cycling, which are believed to be caused by the surrounding graphite buffer effect. Regarding the macroscopic surface state of the electrode, the SiO_x_ blending electrode showed a relatively clean surface state during 500 cycles, while the SiMP blending electrodes showed that large by-products ranging from several micrometers to tens of micrometers were formed on the electrode surface after 250 cycles. Based on these results, the SiO_x_ blending electrode effectively blocks the electrode swelling with the intraparticle and interparticle buffer matrices, which are the SiO_2_ and lithium silicate in SiO_x_ structure and surrounding graphite buffer, respectively.

**Figure 4:**
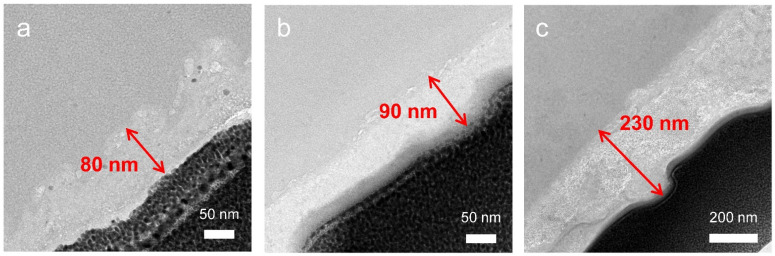
Cross-sectional TEM images of SiO_x_ blending electrode after (**a**) 50 cycles, (**b**) 100 cycles, and (**c**) 250 cycles.

**Figure 5:**
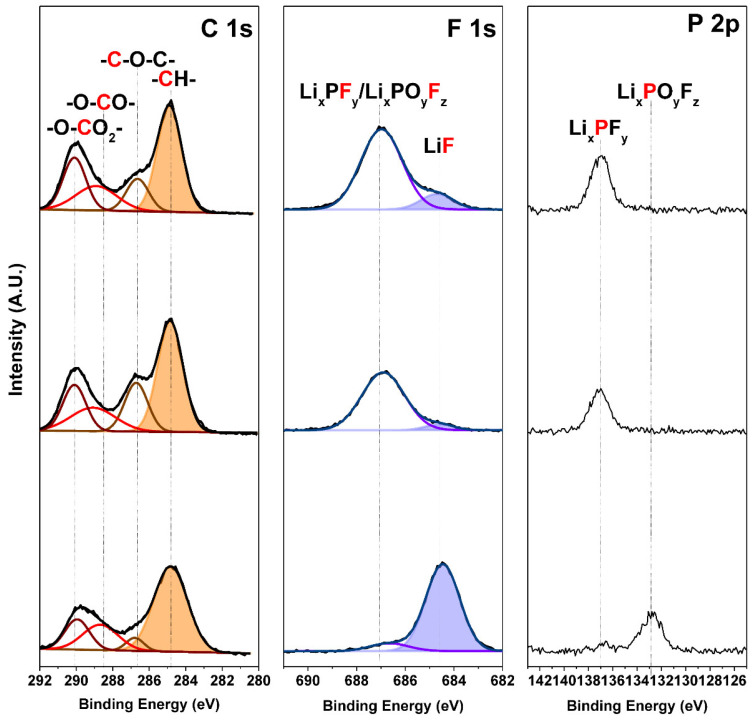
XPS analysis of SiO_x_ blending electrode after 5, 50, and 350 cycles from top to bottom.

To elucidate the cause of the relatively less degradation of the SiO_x_ blending electrode as the cycling progressed in full-cell, we measured cross-sectional high-resolution transmission electron microscopy (HR-TEM) images of SiO_x_ blending anodes after 50, 100, and 250 cycles in Figure 4. The thickness of the SEI layer after 50, 100, and 250 cycles were gradually increased to 80, 90, and 230 nm, respectively. Likewise in the TEM result, electrochemical impedance spectroscopy (EIS) indirectly indicates an increase in SEI by an increase in internal resistance (Figure S5). The right shift of the semi-circle in the Nyquist plot shows increased overpotential in electrolyte due to its depletion, and the increased size of the semi-circle is indicative of increased overpotential due to SEI information. However, although it exhibited gradual thickened SEI, the SEI showed dense and robust characteristics in HR-TEM images, which is contrary to the conventional feature of coarse and loose structure in ordinary silicon anodes [36,37]. This result corresponds with electrode swelling data and the surface state of the electrode in Figure 3 and Figure S4. Figure S6 shows the STEM-BF image and STEM-EDS mapping of the SiO_x_ blending electrode after 250 cycles. The distinct boundary between the SEI layer and SiO_x_ could be observed in the EDS mapping. The SEI mainly contained elements of carbon, phosphorus, and fluorine, since the main components of electrolytes are organic solvents, lithium salts, and additives. We also confirmed the chemical compositions of SEI layers with ex-situ XPS analysis in Figure 5. In the C 1s peaks, we could observe carbon bonding of -O-C-O_2_-, -C-O-C-, and CH_x_. There are no large variations in C 1s peaks [38,39]. In the F 1s peaks, we could observe relative amounts of Li_x_PF_y_ and LiF. In the P 1s peaks, we could observe a relative amount of Li_x_PF_y_ and Li_x_PO_y_F_z_ [40,41]. The LiF peaks are gradually increased after several tens of cycles. The decomposition of LiPF6 or fluoroethylene carbonate (FEC) forms LiF. From these analysis results, we can confirm that the gradually thickened SEI layer in Figure 4 was proven to be fluorine-rich (F-rich). Generally, in the case of Si-based materials, with increasing cycling, the SEI layer becomes thicker or forms by-products, including Si, due to the continuous side reaction of the electrolyte, which reduces the capacity and increases internal resistance, resulting in capacity fading. However, such F-rich layers have been generally ascertained to be robust, strong, and provide stable lithium-ion transport channels, resulting in good electrochemical performances [42]. Therefore, we believe that the superior cyclability of the SiO_x_ blending electrode is strongly related to its uniform formation of the F-rich SEI layer.

## 4. Conclusions

In this study, the superiority of the SiO_x_ and graphite blending electrode compared to the SiMPs and graphite blending electrode was investigated through full-cell-based evaluation. Continuous SEI thickening due to volume change and electrolyte decomposition during cycling may cause capacity to decrease as internal resistance increases. However, in the case of SiO_x_ blending, the F-rich SEI layer formation, which is relatively robust and can serve as a stable transport channel for lithium ions, resulted in superior electrochemical performance compared to the SiMP blending electrode. The strategy of SiO_x_ and graphite blending electrodes shows much more effective physicochemical and electrochemical characteristics in commercial-grade electrodes than SiMPs and graphite blending electrodes, and may clearly suggest a commercial breakthrough direction for Si-based anode materials.

## Data Availability

Not applicable.

## Supplementary Materials

The following supporting information can be downloaded at: https://www.mdpi.com/article/10.3390/nano12121956/s1, Figure S1: (a) Cross-section HR-TEM image and diffraction patterns of SiO_x_. (b) XRD patterns of SiO_x_, commercialized SiO, and SiO_2_; Figure S2: XPS spectra of SiO_x_ surface and core in O 1s and Si 2p; Figure S3: Formation voltage profiles of (a) SiMPs blending and (b) SiO_x_ blending in full cells; Figure S4: Cross-section SEM images of (a–c) SiMPs blending and (d–f) SiO_x_ blending electrodes during 200 cycles in full-cell: (a,d) pristine, (b,e) after 250 cycles, (c,f) after 500 cycles, respectively; Figure S5: EIS analysis of SiO_x_ blending electrode in the full cell. Data were collected after formation, 10, and 50 cycles; Figure S6: Scanning transmission electron microscope (STEM)-bright field (BF) image and STEM-the energy dispersive X-ray spectroscopy (EDS) mapping of SiO_x_ blending electrode after full-cell 250 cycles.
