# Supplementary material for: Micron-Sized SiOx-Graphite Compound as Anode Materials for Commercializable Lithium-Ion Batteries
Source: Nanomaterials (Basel). 2022 Jun 7;12(12):1956. doi: 10.3390/nano12121956 (PMC9230857; doi:10.3390/nano12121956)
Supplement: Supplementary file 1 [file nanomaterials-12-01956-s001.zip › nanomaterials-1757055-supplementary.pdf]

nanomaterials

MDPI

Supplementary Materials

Micron-Sized SiOx-Graphite Compound as Anode Materials for Commercializable Lithium-Ion Batteries

Minki Jo [1,†], Soojin Sim [2,†], Juhyeong Kim [1], Pilgun Oh [3,4], and Yoonkook Son [1,*]

[1] Department of Electric Engineering, Chosun University, 309, Pilmun-daero, Dong-gu, Gwangju 61452, Korea; mkjo@chosun.ac.kr (M.J.); juhyeong@chosun.kr (J.K.)
[2] Department of Energy Engineering, School of Energy and Chemical Engineering, Ulsan National Institute of Science and Technology (UNIST), Ulsan 44919, Korea; thegreatest25@gmail.com
[3] Department of Smart Green Technology Engineering, Pukyong National University, Busan 485471, Korea; poh@pknu.ac.kr
[4] Department of Nanotechnology Engineering, Pukyong National University, Busan 485471, Korea
* Correspondence: y_son@chosun.ac.kr
† These authors contributed equally to this work.

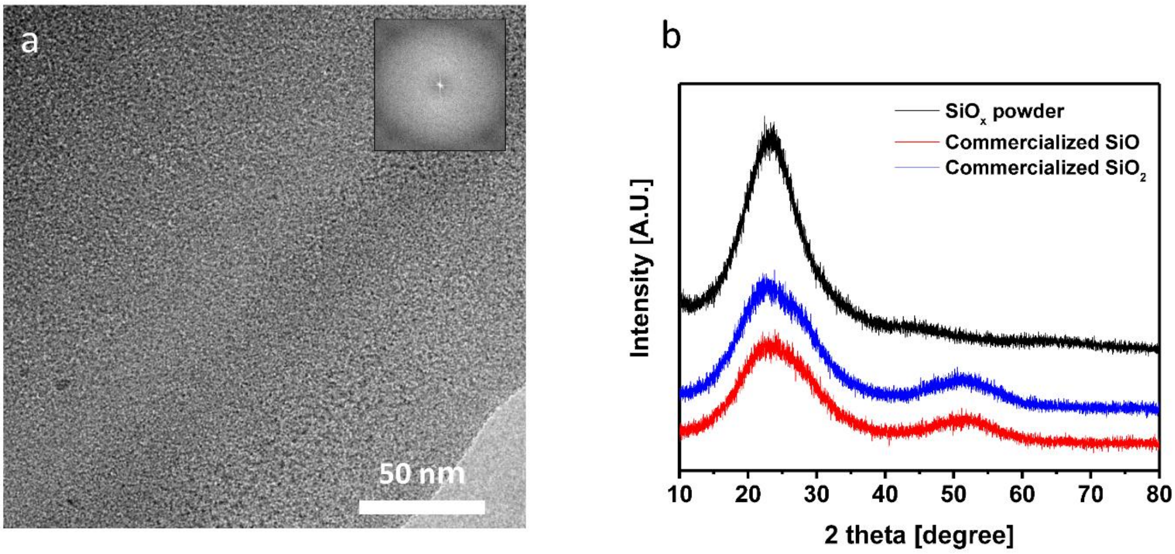

Figure S1. (**a**) Cross-section HR-TEM image and diffraction patterns of SiO_x. (**b**) XRD patterns of SiO_x, commercialized SiO, and SiO_2.

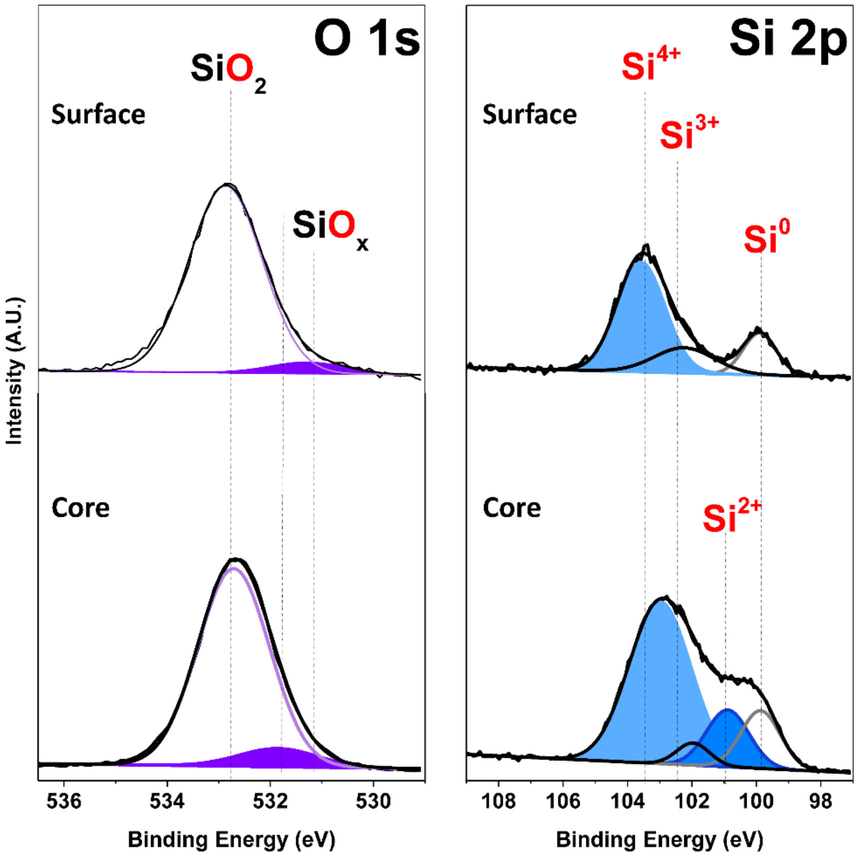

Figure S2. XPS spectra of SiO_x surface and core in O 1s and Si 2p.

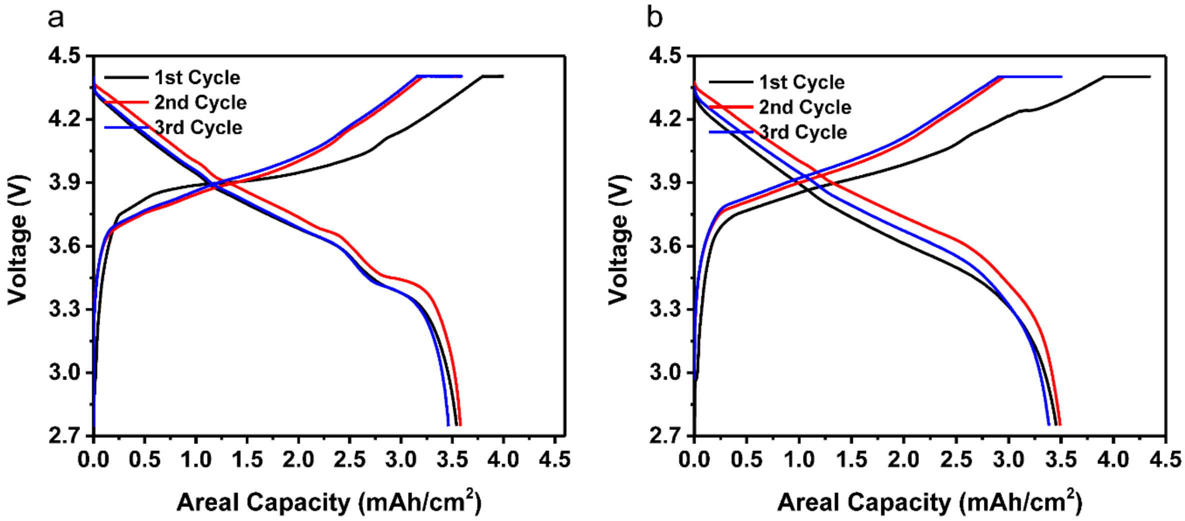

Figure S3. Formation voltage profiles of (**a**) SiMPs blending and (**b**) SiO_x blending in full cells.

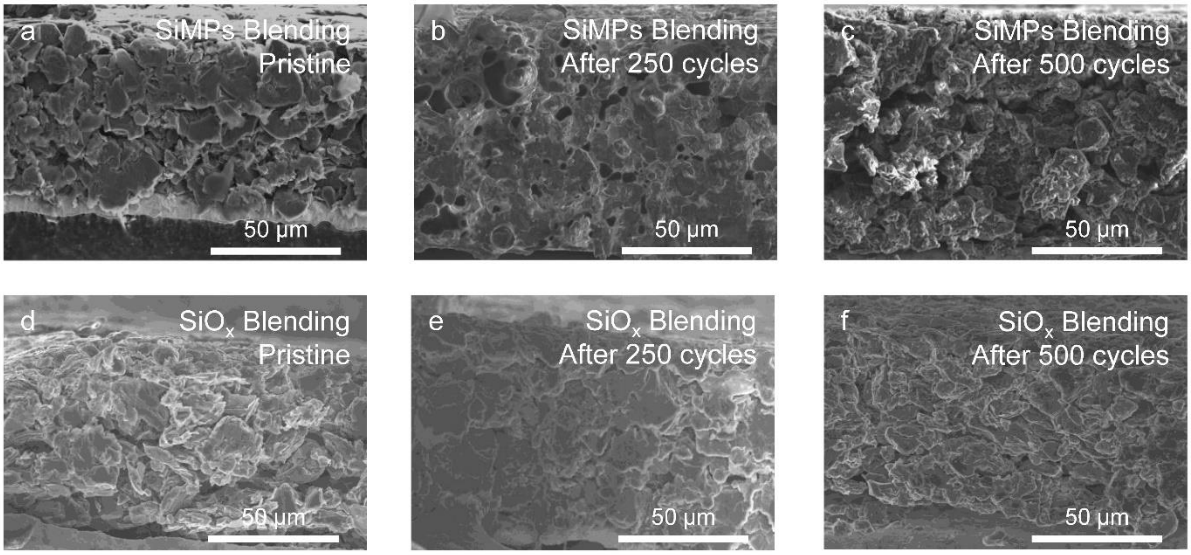

Figure S4. Cross-section SEM images of (**a**–**c**) SiMPs blending and (**d**–**f**) SiOx blending electrodes during 200 cycles in full-cell: (**a**,**d**) pristine, (**b**,**e**) after 250 cycles (**c**,**f**) after 500 cycles, respectively.

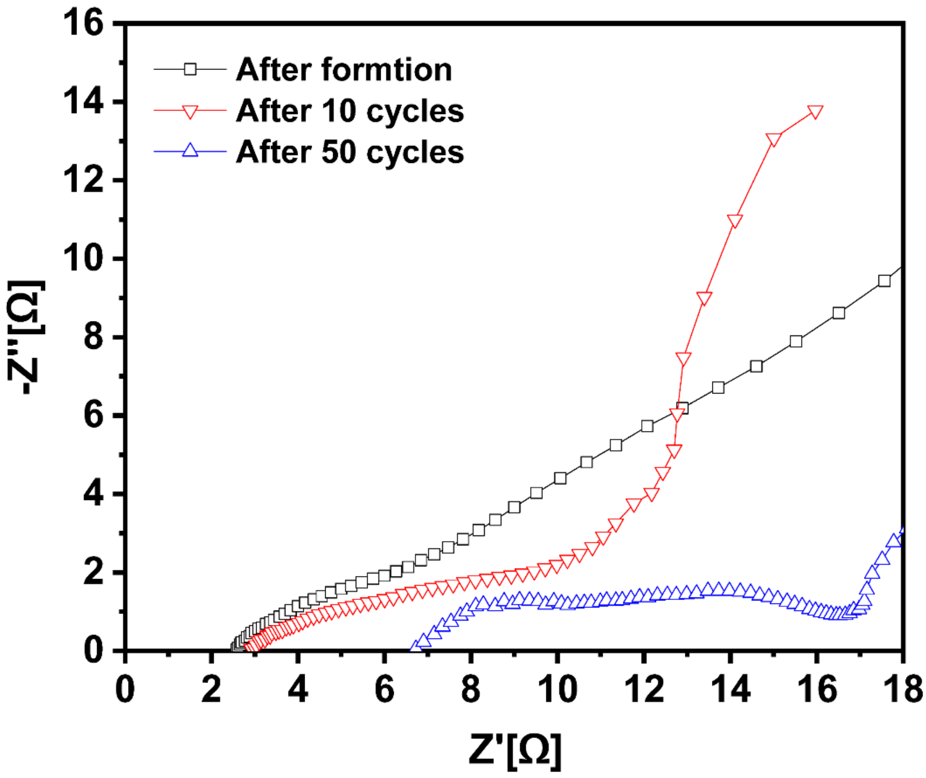

Figure S5. EIS analysis of SiO_x blending electrode in the full cell. Data were collected after formation, 10, and 50 cycles.

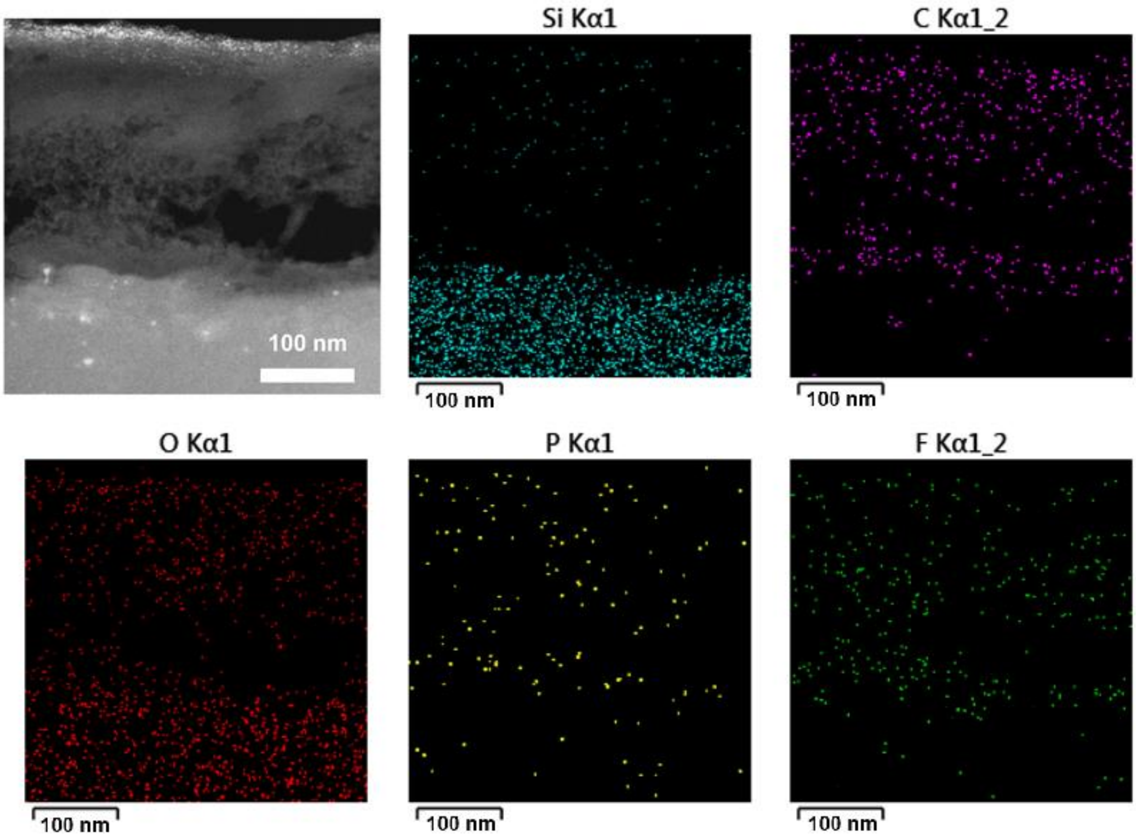

Figure S6. Scanning transmission electron microscope (STEM)-bright field (BF) image and STEM-the energy dispersive X-ray spectroscopy (EDS) mapping of SiO_x blending electrode after full cell 250 cycles.
